# MicroRNA-10 Family Promotes the Epithelial-to-Mesenchymal Transition in Renal Fibrosis by the PTEN/Akt Pathway

**DOI:** 10.3390/cimb44120413

**Published:** 2022-12-02

**Authors:** Chaokun Wang, Yichen Shuai, Chuan Zhao, Fengrui Yang, Weilian Su, Zhifen Ning, Guoxia Li

**Affiliations:** Department of Pharmacology, School of Basic Medical Sciences, Tianjin Medical University, Tianjin 300070, China

**Keywords:** Akt pathway, epithelial-mesenchymal transition, geneknockoutmice, microRNA-10, PTEN, renal fibrosis

## Abstract

Renal fibrosis (RF) is a common reason for renal failure, and epithelial-mesenchymal transition (EMT) is a vital mechanism that promotes the development of RF. It is known that microRNA-10 (miR-10) plays an important role in cancer EMT; however, whether it takes part in the EMT process of RF remains unclear. Therefore, we established an in vivo model of unilateral ureteral obstruction (UUO), and an in vitro model using TGF-β1, to investigate whether and how miR-10a and miR-10b take part in the EMT of RF. In addition, the combinatorial effects of miR-10a and miR-10b were assessed. We discovered that miR-10a and miR-10b are overexpressed in UUO mice, and miR-10a, miR-10b, and miRs-10a/10b knockout attenuated RF and EMT in UUO-treated mouse kidneys. Moreover, miR-10a and miR-10b overexpression combinatorially promoted RF and EMT in TGF-β1-treated HK-2 cells. Inhibiting miR-10a and miR-10b attenuated RF and EMT induced by TGF-β1. Mechanistically, miR-10a and miR-10b suppressed PTEN expression by binding to its mRNA3′-UTR and promoting the Akt pathway. Moreover, PTEN overexpression reduced miR-10a and miR-10b effects on Akt phosphorylation (p-Akt), RF, and EMT in HK-2 cells treated with TGF-β1. Taken together, miR-10a and miR-10b act combinatorially to negatively regulate PTEN, thereby activating the Akt pathway and promoting the EMT process, which exacerbates RF progression.

## 1. Introduction

Renal fibrosis (RF) is a hallmark of renal function deterioration and the final manifestation of a wide variety of renal diseases [1]. The pathological changes in RF are reflected by the destruction and loss of renal tissue structures, including glomeruli, renal tubules, and interstitium, as well as the excessive accumulation of the extracellular matrix (ECM) [2].

Epithelial-mesenchymal transition (EMT) in renal tubular epithelial cells is a key pathogenic mechanism of renal fibrosis (RF) [3]. Renal tubular EMT is a phenotype in which these cells lose their epithelial attributes and acquire the characteristics of mesenchymal cells, such as myofibroblasts. EMT is characterized by the decrease in epithelial cell markers, such as E-cadherin, and the increase in mesenchymal cell markers, such as vimentin and α-SMA [4]. Inhibiting EMT progression in renal tubules may alleviate RF. In order to develop new therapeutic strategies, it is imperative to explore the potential mechanism of EMT in RF.

MicroRNAs (miRNAs) are small non-coding RNAs with approximately 22 nucleotides that repress the translation of target mRNAs by interacting with their 3′-untranslated regions (3′-UTR) [5,6,7]. Additionally, miRNAs are expressed across all tissues, and miRNAs enriched in certain tissues regulate that tissue’s homeostasis [8]. The aberrant regulation of miRNAs expressed in the kidney results in renal dysfunction [9]. A substantial body of evidence indicates that miRNAs are involved in the pathophysiology of the kidney. The miRNAs that have been investigated in AKI include miR-21, which has an anti-apoptotic role [10], and miR-214, which regulates mitochondrial dynamics [11,12]. Moreover, miR-146a protects against inflammation and fibrosis, and is downregulated in diabetic kidney diseases [13]. As members of the miR-10 family, miR-10a and miR-10b are associated with EMT in tumor metastasis and organ fibrosis. Individually, miR-10a promotes EMT in pancreatic cancer and esophageal squamous cell carcinoma [14,15], and miR-10b inhibits breast cancer EMT via CADM2 and induces lung cancer EMT via TAZ [16,17]. Both miR-10a and miR-10b are upregulated in hepatic fibrosis [18,19]. However, their individual and combinatorial functions in the EMT of RF development remain unclear. In particular, research with gene knockout animal models is lacking.

TGF-β1 is the most important inducer of EMT during embryogenesis, fibrosis, and cancer [20]. As a pro-renal fibrotic cytokine, TGF-β1 can promote ECM production and the transdifferentiation of tubular epithelial cells to fibroblasts in HK-2 cells [21]. The unilateral ureteral obstruction (UUO) mouse model is broadly used to simulate RF [22]. We performed UUO surgery in mice to induce symptoms in vivo, and used TGF-β1 to induce HK-2 cell EMT in vitro.

In this study, miR-10a and miR-10b expression was examined in a UUO model, and in HK-2 cells treated with TGF-β1. We investigated the roles and underlying mechanisms of miR-10a and miR-10b in EMT and RF induced by TGF-β1. In addition, their combinatorial effect is discussed. These results were confirmed in the miR-10a^−/−^, miR-10b^−/−^, and miR-10a^−/−^10b^−/−^ knockout UUO models. Based on these observations, miR-10a and miR-10b may be novel targets to block the EMT of RF.

## 2. Materials and Methods

### 2.1. Knockout Mice

The miR-10a knockout (miR-10a^−/−^) mice were obtained from BRL Medicine (Shanghai, China). The miR-10b knockout (miR-10b^−/−^) mice were obtained from WeishangLide Biotechnology Company (Beijing, China). The miR-10a^−/−^ mice were bred with the miR-10b^−/−^ mice to generate miR-10a^−/−^10b^−/−^ mice. All mice had a C57BL/6 background. To confirm knockout, tail DNA samples were genotyped using the following primer pairs:

miR-10a-PCR-S: 5′-CCAAGAACGGACCCACAGT-3′
miR-10a-PCR-A: 5′-AGTGAACAAGGACCCAAGC-3′
miR-10b-PCR-F: 5′-CCAGAAAGGTAAATGCTCG-3′
miR-10b-PCR-R: 5′-ATGAGTGTGGGCAATGTG-3′

### 2.2. Animal Models and Groups

All animal experiments were conducted in strict accordance with institutional guidelines and approved by the Animal Ethics Council of Tianjin Medical University (Doc. No. TMUaMEC 2022005). All mice were maintained at 22 °C and 40–60% humidity. The mice were allowed access to food and water ad libitum. Alternating light/dark cycles were set at 12 h intervals. Eight-week-old male mice (20–23 g) of all genotypes were anesthetized with an intraperitoneal injection of chloral hydrate. Mice were subjected to a left lateral incision to expose the kidney, and the left ureter was doubleligated with 4–0 silk. The right kidney was also exposed, but without ligation of the right ureter. After 7 or 14 days, both left and right kidneys from mice were collected for experimental analysis. The left kidneys were defined as the UUO group while the right kidneys were defined as the sham-operated group.

### 2.3. Cell Culture and Cell Transfection

HEK293T and HK-2 cells were supplied by BNFUTURE. The cells were cultured in a DMEM medium (Gaithersburg, MD, USA) containing 10% fetal bovine serum (HyClon, UT, USA), 100 µg/mL streptomycin, and 100 IU/mL penicillin. All cells were cultured in 5% CO_2_ at 37 °C. All transfection experiments were carried out using the Lipofectamine 2000 transfection reagent (Invitrogen, Carlsbad, CA, USA), following the manufacturer’s instructions. To induce EMT, HK-2 cells were seeded into 12-well plates. They were cultured in serum-free medium for 4 h, then cultured in complete culture medium with PBS or 20 ng/mL TGF-β1 (Invitrogen, USA) for 6 h. RNA expression was evaluated by RT-qPCR 36 h after transfection, and protein expression was evaluated by Western blot 48 h after transfection.

### 2.4. Plasmid Preparation and Oligonucleotide Synthesis

A fragment containing the sequences of pre-miR-10a and pre-miR-10b was cloned into the pcDNA3-vector as an overexpression plasmid (pcDNA3-vector/pre-miR-10a, pcDNA3-vector/pre-miR-10b). The overexpression plasmid of PTEN (pcDNA3-vector/PTEN) and its negative control (Vector), and shRNA specifically targeting PTEN (pSilencer 2.1/shR-PTEN) and its negative control (shR-NC) plasmids, were generously provided by the Tianjin Medical University Basic Medical Research Center and confirmed by sequencing. Fragments containing binding sites and the mutation sites of miR-10a/10b-5p and PTEN were synthesized and ligated into pcDNA3-EGFP to construct the reporter vectors (pcDNA3 EGFP/PTEN-3′-UTR-WT/MUT). Primer and oligonucleotide sequences are listed in Table 1.

### 2.5. HE and Masson Staining

Paraffin-embedded kidney sections were prepared as previously described [23]. HE and Masson staining were used to observe pathological changes. Masson’s trichrome-stained sections were examined under an optical microscope, and images were taken at 100× and 200× magnification (Olympus, Tokyo, Japan). Three fields from the whole section were selected, and the blue-stained area ratio was calculated as the percentage of the total positive area/total analysis area.

### 2.6. EGFP Fluorescent Reporter Assay

The regulation of PTEN expression by miR-10a and miR-10b was evaluated using an EGFP fluorescent reporter assay. HEK293T cells were seeded into 12-well plates. After 24 h, they were transfected with pre-miR-10a, pre-miR-10b, miR-NC, PTEN-3′-UTR-WT, and PTEN-3′-UTR-MUT using Lipofectamine 2000. After 48 h, cells were prepared for fluorescence measurement and Western blot analysis.

### 2.7. Western Blots

Proteins were separated on 10% SDS polyacrylamide gels by electrophoresis, and transferred to PVDF membranes. The membranes were immersed in 5% milk, then incubated at 4 °C with primary antibodies for 12 h. The antibodies were EGFP (Immunoway, YM1515; 1:1000), PTEN (Immunoway, YT3894; 1:2000), α-SMA (Abcam, ab7817; 1:500), E-cadherin (Immunoway, YM3353, 1:500), vimentin (Immunoway, YT4879; 1:1000), collagen-1 (Affinity, AF7001; 1:200), fibronectin (Immunoway, YT1733; 1:200), p-Akt (Immunoway, YP0864; 1:1000), Akt (Immunoway, YT0185; 1:1500) and β-actin (Abcam, ab8226; 1:10,000). After washing with TBST, the membranes were incubated with goat anti-rabbit IgG (Affinity, S0001, 1:10,000) for 2 h at 25 °C, washed again with TBST, and then exposed to X-ray film in the dark. The expression of all proteins was standardized using β-actin as an internal reference protein.

### 2.8. Reverse Transcription-Quantitative PCR 

TRIzol reagent (Invitrogen, Carlsbad, CA, USA) was used to isolate total RNAs from the cell and tissue samples. RNA was reverse-transcribed into cDNA using M-MLV (Promega, Madison, WI, USA). SYBR Premix ExTaq (TaKaRa, Dalian, China) was used for RT-qPCR experiments. The relative mRNA and miRNA expression levels were normalized to β-actin and U6, which were used as internal controls. Relative gene expressions were obtained using the 2−ΔΔCt method. All primer sequences used for RT-qPCR are listed (Table 2).

### 2.9. Statistical Analysis 

Data are presented as mean ± SD. A two-tailed Student’s *t*-test was used to compare the two groups. Statistical significance was set at *p* < 0.05. Experiments were repeated at least three times.

## 3. Results

### 3.1. Expression of miR-10a and miR-10b Was Upregulated in UUO-Induced Mouse Kidneys

**Figure 1:**
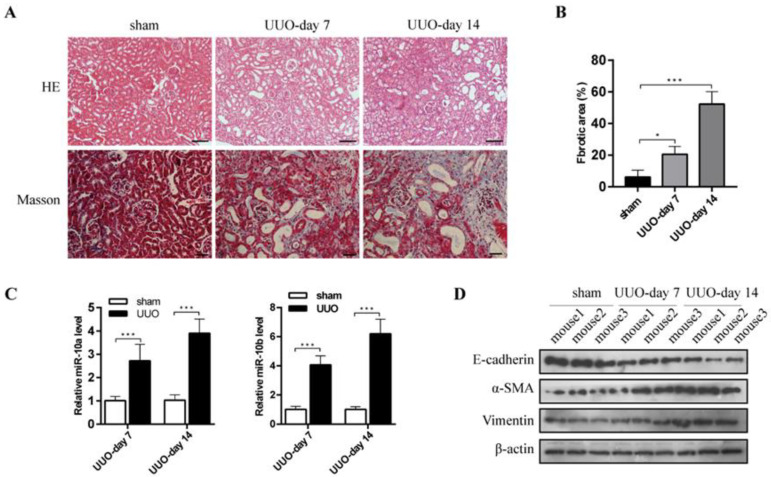
The expression of miR-10a and miR-10b were upregulated in UUO-induced mice kidneys. (**A**) Representative images of HE (image scale bars, 50 µm) and Masson staining (image scale bars, 20 µm) in kidney sections from UUO-induced WT mice for 7 and 14 days. Sham group was used as a control. (**B**) Quantitative analysis of the blue staining area of fibrosis. The sham group was used as a control. (**C**) RT-qPCR analysis for miR-10a and miR-10b in mouse kidneys with/without UUO-induced RF. (**D**) Western blot for E-cadherin, α-SMA, and vimentin in mouse kidneys with/without UUO-induced RF. * *p* < 0.05, *** *p* < 0.001.

To investigate the correlation between miR-10 and RF, we established a mouse model with UUO. The HE and Masson staining of the mouse kidney tissues showed that the UUO mice had tubular atrophy and a widened interstitial area compared with the sham group. There were many blue-stained collagen fibers mutually interwoven and deposited in the renal interstitium. The fibrotic process increased with time (Figure 1A,B). During RF progression in the UUO mice, the RT-qPCR results showed miR-10a had a 2.69- and 3.81-fold increase in the UUO group on day seven and day fourteen after UUO, respectively, and miR-10b showed a 4.00- and 6.11-fold increase, respectively, compared to the sham group (Figure 1C). In addition, E-cadherin expression notably decreased, and α-SMA and vimentin expression notably increased with time in UUO kidney tissue, compared with the sham group (Figure 1D). These results suggested that miR-10a and miR-10b upregulation is related to RF in a time-dependent manner, and confirm EMT involvement in RF progression.

### 3.2. The Absence of miR-10a and miR-10b Mitigated UUO-Induced RF and EMT

**Figure 2:**
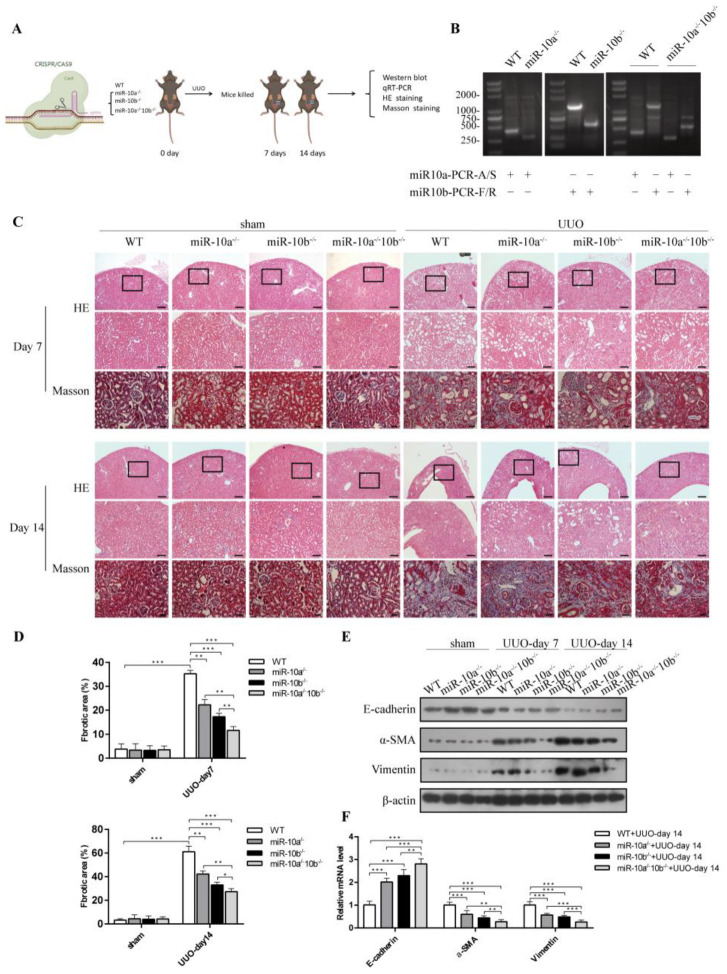
The absence of miR-10a and miR-10b mitigated UUO-induced RF and EMT. (**A**) Schematic diagram of animal experiment process. (**B**) Genotypic identification of knockout mice. (**C**) Representative images of HE (superincumbent image scale bars, 100 µm; lower image scale bars, 50 µm) and Masson staining (image scale bars, 20 µm) in mouse kidneys treated with UUO for 7 and 14 days. Contralateral kidney was used as control. (**D**) Quantitative analysis of blue staining area of fibrosis. At least three random fields were taken from each kidney. (**E**) Western blot for E-cadherin, α-SMA, and vimentin in kidneys. (**F**) RT-qPCR analysis for E-cadherin, α-SMA, and vimentin in mouse kidneys treated with UUO for 14 days. * *p* < 0.05, ** *p* < 0.01, *** *p* < 0.001.

EMT plays a vital role in RF pathogenesis [3]. To explore the roles of miR-10a and miR-10b in RF and EMT, the WT, miR-10a^−/−^, miR-10b^−/−^ and miR-10a^−/−^10b^−/−^ mice were subjected to UUO surgery (Figure 2A). Genotype screening confirmed the lack of miR-10a/10b in the knockout strains of the miR-10a^−/−^, miR-10b^−/−^, and miR-10a^−/−^10b^−/−^ mice (Figure 2B). HE and Masson staining analysis showed tubular atrophy and a widened interstitial area; there were many blue-stained collagen fibers mutually interwoven and deposited in the renal interstitium in the UUO-treated kidneys compared to the contralateral kidneys (Figure 2C,D). We also measured the expression of RF (vimentin and collagen-1) and EMT markers (E-cadherin, α-SMA, and vimentin). Compared with the contralateral kidney, fibronectin, collagen-1, α-SMA, and vimentin expression was upregulated, whereas E-cadherin was downregulated in the UUO-treated kidneys; the degree of RF and EMT was more severe on day fourteen than on day seven (Figure 2E). These results demonstrated that our mouse model was successfully established. As shown in Figure 2C–F, both on day seven and day fourteen after UUO, compared with the UUO-treated WT group, miR-10a and miR-10b knockout attenuated UUO-induced pathological changes of fibrosis, downregulated fibronectin and collagen-1 expression, and downregulated α-SMA and vimentin expression, whereas it upregulated E-cadherin expression. EMT marker (E-cadherin, α-SMA, and vimentin) mRNA expression of UUO on day fourteen was consistent with the above results. In three kinds of gene knockout mice groups, the degree of the fibrotic lesions was the least severe, and the fibronectin, collagen-1, α-SMA, and vimentin expression levels were the lowest, while the E-cadherin expression level was the highest in UUO-treated miR-10a^−/−^10b^−/−^ mice. Collectively, miR-10a and miR-10b knockout combinatorially disrupted RF development and EMT in vivo.

### 3.3. miR-10a and miR-10b Overexpression Promoted TGF-β1-Induced RF and EMT

**Figure 3:**
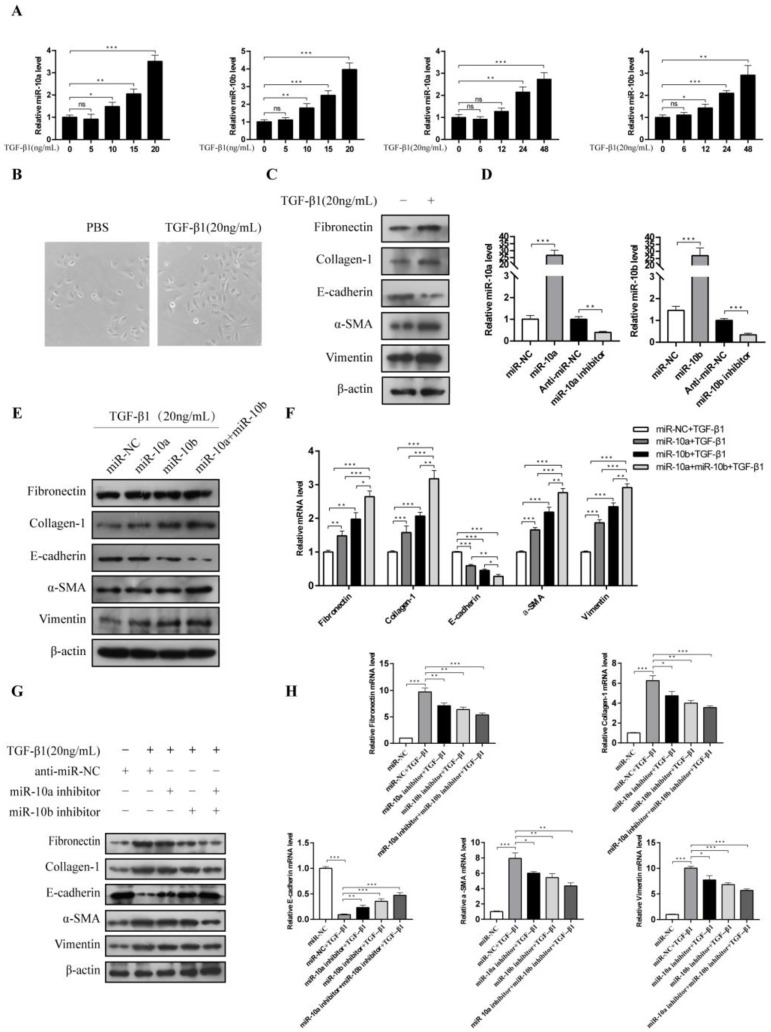
The miR-10a and miR-10b overexpression promoted TGF-β1-induced RF and EMT. (**A**) RT-qPCR analysis for miR-10a and miR-10b expression in the HK-2 cells treated with different concentrations (ng/mL) and time (h) of TGF-β1. (**B**) TGF-β1 changed epithelial cell morphology. (**C**) Western blot for fibronectin, collagen-1, E-cadherin, α-SMA, and vimentin expression in HK-2 cells induced or not induced by TGF-β1. (**D**) RT-qPCR analysis for miR-10a and miR-10b expression in the HK-2 cells. (**E**,**F**) Western blot and RT-qPCR analyses for fibronectin, collagen-1, E-cadherin, α-SMA, and vimentin expression in HK-2 cells after transfection with miR-10a and miR-10b or control, followed by treatment with 20 ng/mL TGF-β1. (**G**,**H**) Western blot and RT-qPCR analyses for fibronectin, collagen-1, E-cadherin, α-SMA, and vimentin expression in HK-2 cells after transfection with miR-10a, miR-10b, or control inhibitors, followed by treatment with 20 ng/mL TGF-β1. * *p* < 0.05, ** *p* < 0.01, *** *p* < 0.001. ns: not significant.

TGF-β1 is a pro-fibrotic cytokine in RF that can induce HK-2 cells to undergo EMT [20], which we confirmed in our dose-finding studies, where we monitored miR-10a and miR-10b levels. The miR-10a and miR-10b expressions were upregulated in a dose- and time-dependent manner, compared to the control group (Figure 3A). We also determined the dosage of TGF-β1 (20 ng/mL) to be used, as the EMT change of the HK-2 cells was the most obvious. TGF-β1 changed the epithelial cell morphology from cobblestone to elongated and hypertrophic shapes with cell-cell detachment (Figure 3B). The RF-associated proteins (fibronectin and collagen-1) and EMT-associated proteins (α-SMA and vimentin) were notably increased, whereas the E-cadherin levels were reduced by TGF-β1 stimulation (Figure 3C), verifying that TGF-β1 can induce RF and EMT. The HK-2 cells were transfected with miR-10a, miR-10b, and both miR-10a and miR-10b. The RT-qPCR results demonstrated that both miR-10a and miR-10b expression was enhanced in the HK-2 cells after transfection compared with the control group (Figure 3D). Western blot and RT-qPCR analyses showed that the protein and mRNA levels of fibronectin, collagen-1, α-SMA, and vimentin were significantly increased, and E-cadherin was significantly decreased in the miR-10a- and miR-10b-overexpressing HK-2 cells treated with TGF-β1. Both miR-10a and miR-10b overexpression had a stronger inhibitory effect on EMT and RF than overexpression of either miR-10a or miR-10b alone (Figure 3E,F). We also wanted to figure out whether miR-10a and miR-10b compensate for each other. As Figure S1 shows, compared with the control group, the reduction of miR-10a and miR-10b attenuated TGFβ1-induced EMT in HK-2 cells, promoted PTEN expression, and downregulated α-SMA and vimentin expression, whereas it up-regulated E-cadherin and PTEN expression. Overexpressing miR-10b rescued the miR-10a effects on E-cadherin, α-SMA, vimentin, and PTEN levels. Meanwhile, overexpressing miR-10a rescued the miR-10b effects on the E-cadherin, α-SMA, vimentin, and PTEN levels. In addition, Western blot and RT-qPCR analyses showed that miR-10a and miR-10b inhibitors significantly attenuated the TGF-β1-induced increase of fibronectin, collagen-1, α-SMA and vimentin, and the decrease of E-cadherin (Figure 3G,H). These findings indicated that miR-10a and miR-10b play a combinatorial and mutually compensating role in promoting RF and EMT, and that miR-10a and miR-10b may be important factors for TGF-β1 to promote RF and EMT.

### 3.4. miR-10a and miR-10b Targeted PTEN to Up-Regulate PTEN Expression

**Figure 4:**
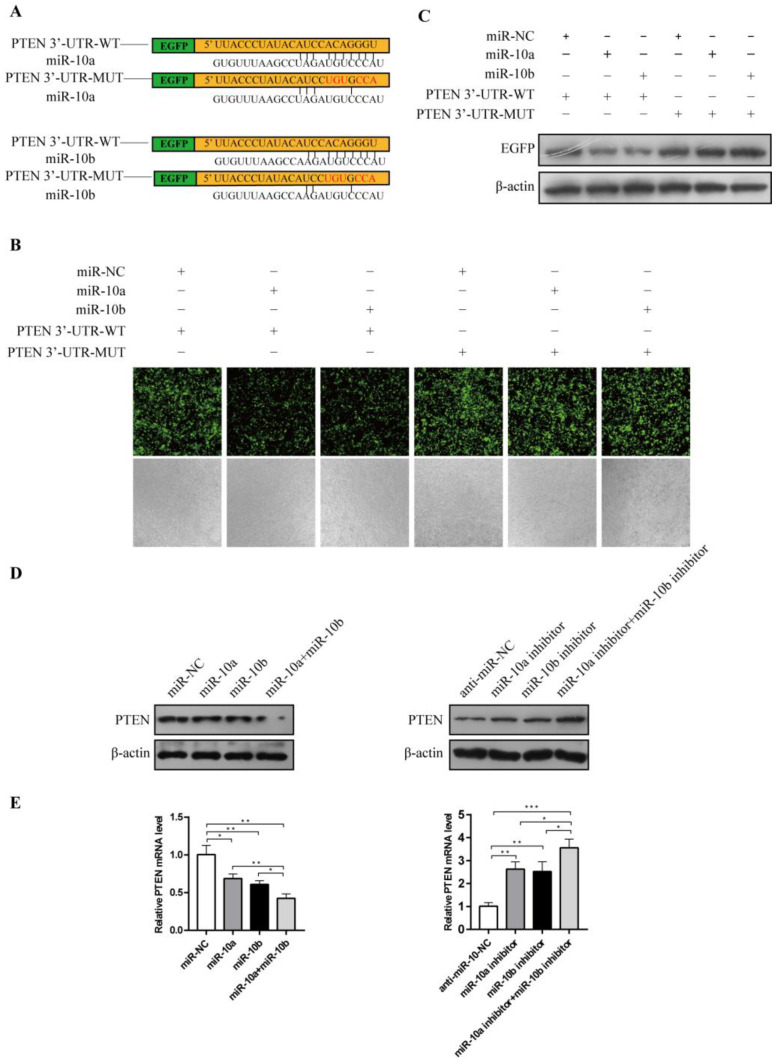
Both miR-10a and miR-10b targeted the PTEN to upregulate PTEN expression. (**A**) TargetScan predicted PTEN-3′-UTR for miR-10a and miR-10b binding. (**B**) Transfection of miRNA-NC, miRNA-10a, or miRNA-10b in an EGFP fluorescent reporter assay using different PTEN-3′-UTR mutational states. (**C**) Western blot for EGFP expression following transfection. (**D**) Western blot for PTEN expression following miR-NC, miRNA-10a, miR-10b, or both miR-10a and miR-10b transfection. (**E**) RT-qPCR analysis for PTEN mRNA following miR-10a, miR-10b, or both miR-10a and miR-10b inhibitor transfection. * *p* < 0.05, ** *p* < 0.01, *** *p* < 0.001.

Because both miR-10a and miR-10b promote RF and EMT, we hypothesized that these miRNAs may promote these processes by the same mechanism. TargetScan was used to predict the potential target mRNAs of miR-10a and miR-10b, and the results showed complementary sequences between these miRNAs and the 3′-UTR of PTEN mRNA (Figure 4A). We investigated whether PTEN was a potential target of miR-10a and miR-10b. Western blot and the EGFP fluorescent reporter assay showed that EGFP expression was decreased when co-transfected with miR-10a, miR-10b, and PTEN-3′-UTR-WT. However, these effects disappeared when the complementary sequences were mutated (Figure 4B,C). Western blot and RT-qPCR analyses showed that the protein and mRNA levels of PTEN were significantly decreased in the HK-2 cells transfected with miR-10a, miR-10b, and both miR-10a and miR-10b. The overexpression of both miR-10a and miR-10b had a stronger inhibitory effect on PTEN than the overexpression of either miR-10a or miR-10b alone. However, the opposite effect occurred after transfection with anti-miR-10a, anti-miR-10b, and both anti-miR-10a and anti-miR-10b (Figure 4D,E). These results suggested that miR-10a and miR-10b combinatorially suppressed PTEN expression by binding to the PTEN mRNA 3′-UTR.

### 3.5. miR-10a and miR-10b Promoted RF and EMT through the PTEN/Akt Pathway

**Figure 5:**
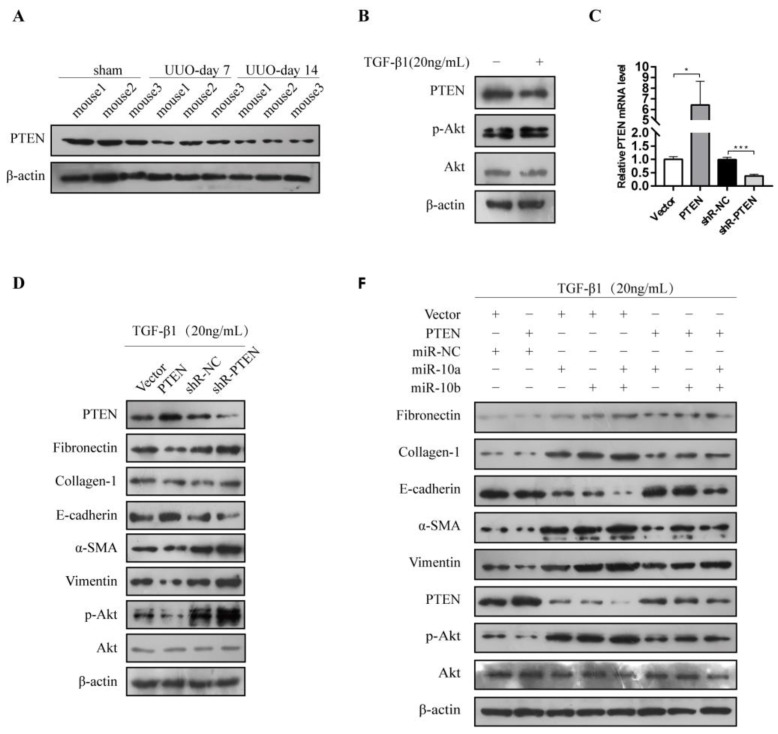

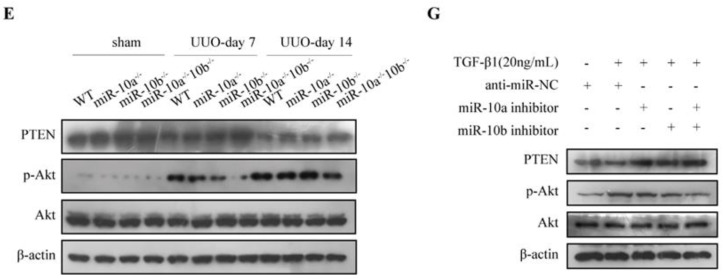
Both miR-10a and miR-10b promoted RF and EMT through the PTEN/Akt pathway. (**A**) Western blot for PTEN expression in UUO mice. (**B**) Western blot for PTEN and p-Akt expression in HK-2 cells incubated with or without 20 ng/mL TGF-β1. (**C**) RT-qPCR analysis for PTEN mRNA expression in HK-2 cells. (**D**) Western blot for fibronectin, collagen-1, E-cadherin, α-SMA, vimentin, PTEN, and p-Akt expression in HK-2 cells after transfecting PTEN-related plasmids followed by incubation with 20 ng/mL TGF-β1. (**E**) Western blot for PTEN and p-Akt expression. (**F**) Western blot for fibronectin, collagen-1, E-cadherin, α-SMA, vimentin, PTEN, and p-Akt expression in HK-2 cells after transfection followed by incubation with 20 ng/mL TGF-β1. (**G**) Western blot for PTEN and p-Akt expression after transfection in HK-2 cells induced or not induced by TGF-β1. * *p* < 0.05, *** *p* < 0.001.

We further explored the mechanism by which miR-10a and miR-10b promote RF and EMT. PTEN negatively regulates p-Akt expression, which is closely related to RF development [24]. We demonstrated that PTEN expression was remarkably decreased in the HK-2 cells treated with TGF-β1 and in the UUO mice (Figure 5A,B). Moreover, p-Akt was also remarkably increased in the TGF-β1-stimulated HK-2 cells (Figure 5B). To further investigate PTEN function, PTEN-silencing plasmids were constructed. RT-qPCR analysis showed that the PTEN mRNA levels were significantly increased and decreased in the PTEN- and shR-PTEN-transfected HK-2 cells, respectively (Figure 5C). PTEN overexpression increased the E-cadherin levels and reduced the fibronectin, collagen-1, α-SMA, vimentin, and p-Akt levels in the HK-2 cells treated with TGF-β1. These results were reversed when PTEN was knocked down (Figure 5D). These results demonstrated that PTEN inhibits RF and EMT by negatively regulating p-Akt. We have already shown that miR-10a and miR-10b overexpression reduces E-cadherin and PTEN, and increases fibronectin, collagen-1, α-SMA, vimentin, and p-Akt (Figure 3E,F). Consistent with these in vitro results, miR-10a and miR-10b knockdown in vivo notably increased PTEN expression and decreased p-Akt (Figure 5E). As Figure 5F shows, overexpressing PTEN abolished the miR-10a and miR-10b effects on E-cadherin, fibronectin, collagen-1, α-SMA, vimentin, and p-Akt levels. This rescue experiment clarified that PTEN antagonizes the fibrotic and EMT-promoting effects of miR-10a and miR-10b in TGF-β1-treated HK-2 cells. In addition, we demonstrated that TGF-β1 inhibits PTEN expression and promotes p-Akt, and the rescue experiments showed that miR-10a and miR-10b inhibitors blocked the PTEN and p-Akt effects induced by TGF-β1 (Figure 5G). These results demonstrated that miR-10a and miR-10b promote RF and EMT induced by TGF-β1 through the PTEN/Akt pathway.

## 4. Discussion

**Figure 6:**
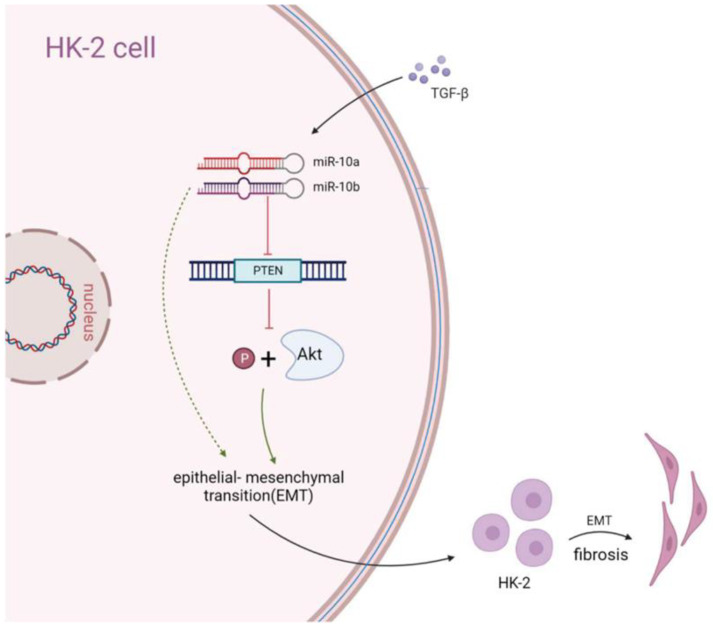
Schematic for miR-10a and miR-10b promoting the EMT of RF through the PTEN/Akt pathway. The black arrow represents the regulatory process, the red arrow represents the inhibitory effect, the green arrow represents the promoting effect and the dotted green arrow represents the indirect effect.

EMT plays a vital role in RF pathogenesis. This study uncovered the role and underlying mechanisms of miR-10a and miR-10b in the EMT process in RF. We clarified the role of the miRs-10a/10b/PTEN/Akt pro-fibrotic axis in the TGF-β1-regulated EMT process of RF (Figure 6). Additionally, we were the first to use and validate the role of miR-10a and miR-10b by using miR-10a^−/−^, miR-10b^−/−^, and miR-10a^−/−^10b^−/−^ mice subjected to UUO. Additionally, the combinatorial effects of miR-10a and miR-10b were demonstrated. These results suggest novel RF treatments that target miR-10a and miR-10b.

Human miR-10a and miR-10b belong to the miR-10 family and originate from different chromosomes: miR-10a is located between the homeobox protein Hox-B4 (HOXB4) and homeobox protein Hox-B5 (HOXB5) genes on chromosome 17, while miR-10b is located in the middle of the homologous box gene cluster, homeobox protein Hox-D10 (HOXD10), on chromosome 2. The primary sequences of miR-10a and miR-10b are identical, except for the twelfth nucleotide [25]. High sequence similarity indicates that miR-10a and miR-10b may play similar roles in pathological processes. This was confirmed by our conclusion that miR-10a and miR-10b promote the RF EMT process and inhibit PTEN activity. However, another study on kidney EMT progression suggested that miR-10a and miR-10b overexpression inhibits EMT in renal cell carcinoma [26,27]. This difference may be due to differences in disease etiology and cell types. With respect to the sequences, the different bases were not in the seed region, thus there was no significant difference in PTEN inhibition between miR-10a and miR-10. However, miR-10b had a stronger impact on the EMT in RF. One explanation is that miR-10a and miR-10b may promote the EMT process through pathways other than inhibiting PTEN. It is worth noting that the combined effect of miR-10a and miR-10b was stronger than that of miR-10a and miR-10b alone, indicating that there is a combinatorial effect between miR-10a and miR-10b.

Our study has identified that miR-10a and miR-10b promote RF and EMT induced by TGF-β1 and UUO. This result contradicts another study that reported that decreasing these miRs aggravated RF in a streptozotocin-induced DKD mouse model [28]. The UUO model we used impairs kidney function by blocking the ureter. The new obstruction may cause a significant increase in ureteral pressure, and hemodynamic changes in the kidney, often resulting in acute renal injury. This leads to dilation of the renal tubule, then flattening, atrophy, infiltration of inflammatory cells, and the formation of fibrosis. Renal fibrosis can also occur in ischemia-reperfusion-induced acute kidney injury [29,30,31]. Diabetic kidney disease (DKD) is a major cause of morbidity, and it often causes chronic kidney damage [32]. The DKD process involves renal hemodynamic changes, oxidative stress, inflammation, hypoxia, and an overactive renin-angiotensin-aldosterone system (RAAS). Renal fibrosis, one of the cardinal histological features of DKD, also plays a key role in DKD [33]. In addition, previous studies have reported inconsistent or contradictory findings regarding whether miR-10a and miR-10b promote kidney injury. Previous studies have demonstrated that miR-10a significantly promotes acute kidney injury (especially ischemia-reperfusion, which is characterized by severe tubular injury that leads to RF) caused by various causes [34,35,36,37], while miR-10b relatively lacks such data. Conversely, in a high-sugar environment, miR-10a significantly inhibited chronic kidney injury caused by type 2 diabetes [38], and miR-10a expression was increased in a diabetic kidney injury model [39]. These studies suggest that miR-10a and miR-10b may play many roles in renal injury or fibrosis caused by different models or different causes. We speculate that the miR-10 family may promote renal fibrosis caused by acute kidney injury, and inhibit renal fibrosis caused by chronic kidney injury due to a high glucose environment.

Our study found that the miRs-10a/10b/PTEN/Akt pro-fibrotic axis is essential in the TGF-β1-regulated EMT process of RF. TGF-β1 is a pivotal regulator of RF pathogenesis and EMT. Several studies have reported that TGF-β1 exerts its functions partly through upregulating miR-21 and lncRNA-ATB [40,41,42]. TGF-β1 induces TWIST1 expression, which is a critical regulator of EMT [43], and has been shown to induce the expression of miR-10a and miR-10b [44,45]. Our study also demonstrated that TGF-β1 can increase miR-10a and miR-10b expression, but we did not explore the mechanism. Moreover, our experiments showed that TGF-β1 inhibited PTEN expression and enhanced RF, EMT, and p-Akt. The rescue experiments in TGF-β1-treated HK-2 cells showed that using miR-10a and miR-10b inhibitors blocked increases in RF, EMT, and p-Akt, and decreases in PTEN. This suggests that miR-10a and miR-10b are key mediators of RF induced by TGF-β1.

As a protein and lipid phosphatase, PTEN represses the phosphoinositide 3-kinase pathway. PTEN deficiency hyperactivates Akt signaling, which affects many cellular processes, including proliferation, migration, the cell cycle, and apoptosis [46]. Previous research demonstrated that PTEN is expressed differently in RF. PTEN expression is downregulated in several other kidney diseases, and is regulated by the TGF-β1 signaling pathway. Immunohistochemistry revealed that UUO led to a marked reduction in PTEN levels in the tubulointerstitial compartment [47]. Our results are consistent with these studies. In our study, PTEN was notably downregulated and p-Akt was notably upregulated in the UUO mice and in the TGF-β1-treated HK-2 cells. PTEN inhibited RF, EMT, and p-Akt in the HK-2 cells treated with TGF-β1. These results indicate that PTEN’s inhibition of Akt leads to inhibited fibrotic signaling pathways. In the HK-2 cells, miR-10a and miR-10b overexpression significantly reduced the PTEN levels, and increased p-Akt expression. Conversely, the deletion of miR-10a, miR-10b, and miRs-10a/10b in the UUO mice notably increased PTEN expression and reduced p-AKT. In addition, miR-10a and miR-10b downregulated PTEN by binding to the 3′-UTR of PTEN. This result has also been demonstrated in previous studies [48,49,50,51]. It is worth noting that although PTEN overexpression abolished the effects of miR-10 on RF and EMT, the expression levels of EMT- and RF-related proteins were still much higher than those in the control mice. These results indicate that PTEN is an important mediating factor in the process through which miR-10a and miR-10b promote RF and EMT, but these miRNAs are not the sole regulatory factors of PTEN.

In addition to the miR-10 family, the aberrant expression of other miRNAs perturbs signaling pathways that lead to the progression of kidney fibrosis. Moreover, miR-21 expression increases in the kidneys of mice subjected to unilateral ureteral obstruction (UUO) or ischemic reperfusion injury (IRI), the two well-established animal models of kidney fibrosis. The mechanism for the antifibrotic effects of miR-200 may involve the prevention of EMT [52,53]. The expression of miR-29 is also decreased in mouse models of renal fibrosis [54,55]. Potential target genes of the miR-10 family have also been implicated in renal fibrosis. Growing evidence was obtained regarding the functional roles of BDNF/TrkB signaling in organ and tissue fibrosis [56]. BDNF-mediated TrkB activation has a stabilizing effect on podocyte homeostasis, and has a rescue effect in different podocyte injury models [57]. Recently, the protective role of Vasohibin-1(VASH-1), a negative feedback regulator of angiogenesis, was reported in diabetic nephropathy. The data demonstrated a protective role for endogenous VASH-1 on tubulointerstitial alterations via its regulation of inflammation and fibrosis, and showed the direct antifibrotic effects of VASH-1 on renal fibroblasts by modulating TGF-β1 signaling [58]. Despite many miRNAs and their relevant mRNA targets having been reported to play a role in renal fibrosis, the normal functions of these miRNAs, and a comprehensive list of their biologically relevant mRNA targets, remain unknown. Therefore, this should be investigated thoroughly in future research.

In conclusion, our study illustrates the importance of miR-10a and miR-10b in the EMT process of RF. Our findings provide new insights into the pathogenesis of RF, and suggest that targeting miR-10a and miR-10b may be an effective therapeutic approach for treating RF.

## 5. Conclusions

Both miR-10a and miR-10b act combinatorially to negatively regulate PTEN, thereby activating the Akt pathway and promoting the EMT process, which exacerbates RF progression. Targeting miR-10a and miR-10b may be an effective therapeutic approach for treating RF.

## Figures and Tables

**Table 1:** Primers and oligonucleotides.

**Name**	**Sequence (5′—3′)**
**Primers for expression vectors construction**	
**pre-miR-10a-5p—Forward**	**CATTCGGATCCCAAGAACAGACTCGCAC**
**pre-miR-10a-5p—Reverse**	**GGGAGAATTCGGGGAGAGTTCAGGTAGATG**
**pre-miR-10b-5p—Forward**	**GAAGCTTCCAGAGGTTGTAACGTTGTC**
**pre-miR-10b-5p—Reverse**	**CCGAATTCTGAAGTTTTTGCATCGACCA**
**Oligo nucleotides for making constructs**	
**PTEN-(miR-10a/10b)-UTR-WT-Top**	**GATCCTTACCCTATACATCCACAGGGTTAAGCTTG**
**PTEN-(miR-10a/10b)-UTR-WT-Bot**	**AATTCAAGCTTAACCCTGTGGATGTATAGGGTAAG**
**PTEN-(miR-10a/10b)-UTR-Mut-Top**	**GATCCTTACCCTATACATCCTGTGCCATAAGCTTG**
**PTEN-(miR-10a/10b)-UTR-Mut-Bot**	**AATTCAAGCTTATGGCACAGGATGTATAGGGTAAG**
**Oligonucleotides or genes**	
**anti-miR-NC**	**CAGUACUUUUGUGUAGUACAA**
**miR-10a inhibitor**	**CACAAATTCGGATCTACAGGGTA**
**miR-10b inhibitor**	**CACAAATTCGGTTCTACAGGGTA**

**Name**

**Sequence (5′—3′)**

**Table 2:** Sequences of each primer.

Primer	Sequence (5′—3′)
**miR-10 RT**	**GTCGTATCCAGTGCAGGGTCCGAGGTGCACTGGATACGACAATTTGTG**
**miR-10a-Forward**	**TGCGGTACCCTGTAGATCCGAATTTGTG**
**miR-10b-Forward**	**TGCGGTACCCTGTAGAACCGAATTTGTG**
**U6 RT**	**GTCGTATCCAGTGCAGGGTCCGAGGTATTCGCACTGGATACGACAAAATATGGAAC**
**U6-Forward**	**TGCGGGTGCTCGCTTCGGCAGC**
**U6-Reverse**	**CCAGTGCAGGGTCCGAGGT**
**PTEN-Forward**	**TGGATTCGACTTAGACTTGACCT**
**PTEN-Reverse**	**GGTGGGTTATGGTCTTCAAAAGG**
**E-cad-Forward**	**AAAGGCCCATTTCCTAAAAACCT**
**E-cad-Reverse**	**TGCGTTCTCTATCCAGAGGCT**
**Vimentin-Forward**	**TGCCGTTGAAGCTGCTAACTA**
**Vimentin-Reverse**	**CCAGAGGGAGTGAATCCAGATTA**
**α-SMA-Forward**	**CTATGAGGGCTATGCCTTGCC**
**α-SMA-Reverse**	**GCTCAGCAGTAGTAACGAAGGA**
**β-actin-Forward**	**CGTGACATTAAGGAGAAGCTG**
**β-actin-Reverse**	**CTAGAAGCATTTGCGGTGGAC**

## Data Availability

Not applicable.

## Supplementary Materials

The following supporting information can be downloaded at: https://www.mdpi.com/article/10.3390/cimb44120413/s1, Figure S1: miR-10a and miR-10b have mutually compensating effects.
