# Supplementary figures and images for: MicroRNA-10 Family Promotes the Epithelial-to-Mesenchymal Transition in Renal Fibrosis by the PTEN/Akt Pathway
Source: Curr Issues Mol Biol. 2022 Dec 2;44(12):6059–74. doi: 10.3390/cimb44120413 (PMC9776942; doi:10.3390/cimb44120413)

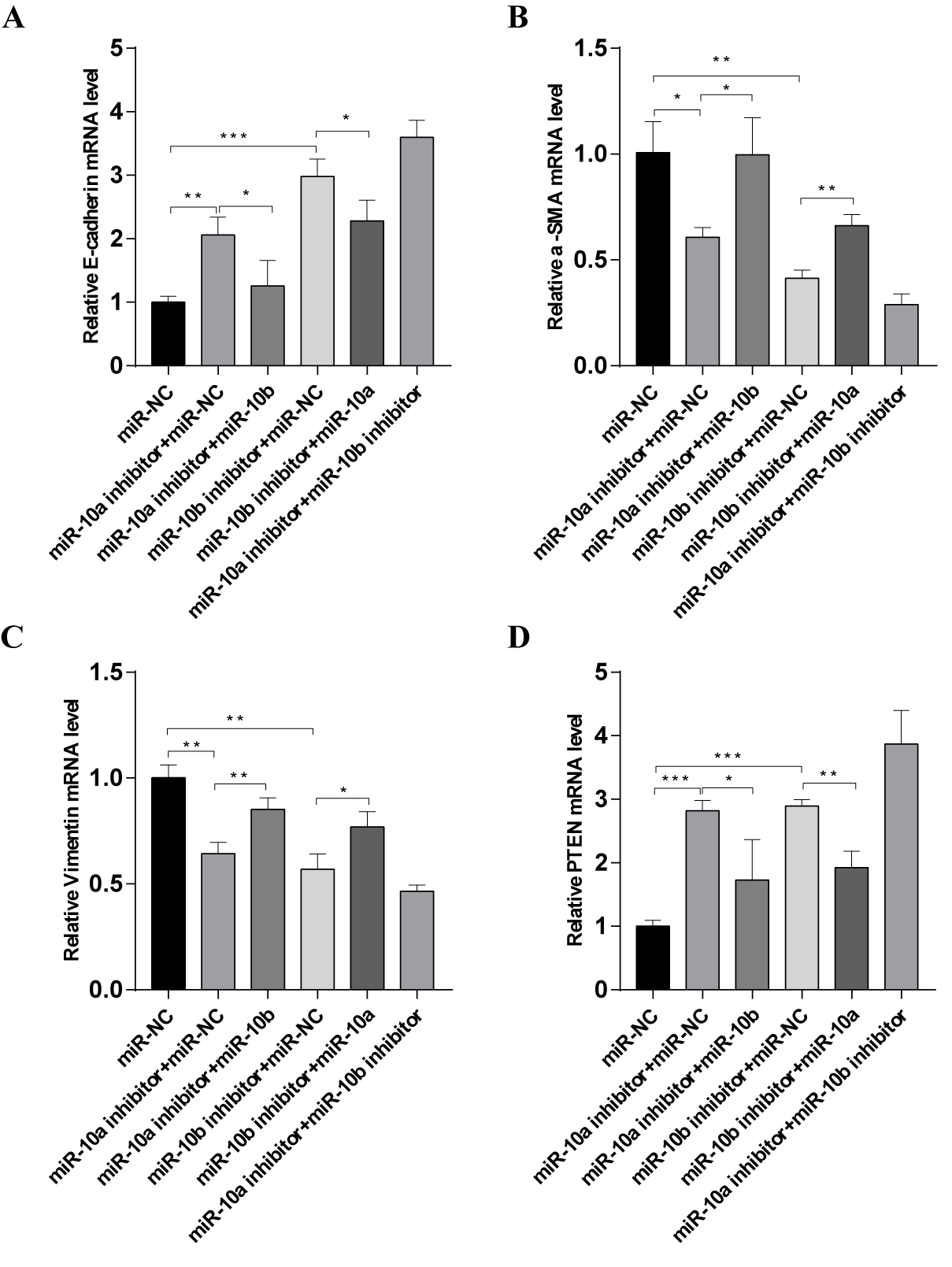

Supplement: Supplementary file 1 [file cimb-44-00413-s001.zip › cimb-2041927-supplementary.tif]
